# Does low infusion pressure microincision cataract surgery (LIPMICS) reduce frequency of post-occlusion breaks?

**DOI:** 10.22336/rjo.2022.27

**Published:** 2022

**Authors:** Hanga Beres, Diego de Ortueta, Benedikt Buehner, Gabor Bernd Scharioth

**Affiliations:** * Aurelios Augenzentrum Recklinghausen, Germany; ** “George Emil Palade” University of Medicine, Pharmacy, Science and Technology of Târgu Mureș; *** University of Szeged, Hungary

**Keywords:** phacoemulsification, phaco fluidics, surge, postocclusion break, target intraocular pressure

## Abstract

**Objective:** To compare the number of surge events and efficacy of phacoemulsification using a near-physiological intraocular pressure (IOP) setting and a standard IOP setting.

**Materials and methods:** The surgical data of patients who underwent phacoemulsification with IOL implantation using the CENTURION Vision System’s Active Fluidics setting and Active Sentry Handpiece (Alcon Laboratories, USA) were analyzed.

**Results:** The study included 181 patients (204 eyes). In Group 1, the IOP was set at 20 mmHg (n=102, 50%), and in Group 2, the IOP was set at 60 mmHg (n=102, 50%). Total case time was significantly lower (p=.036) in Group 1 (0:03:17.20 ± 0:00:34.55 vs. 0:03:28.71 ± 0:00:43.03). There was no statistically significant difference between the mean cumulative dissipated energy (CDE) (7.06 ± 3.20 vs. 7.59 ± 3.26) and mean ultrasound (UJS) time (0:00:36 ± 0:00:12 vs. 0:00:38 ± 0:00:13) between the two groups (p=0.24 and p=0.31, respectively). Active sentry (AS) engaged less often (p<0.001) in Group 1. There was no statistically significant correlation between the CDE and AS activation in Group 1 (p=0.96). A strong statistically significant correlation between the CDE and AS activation (p<0.0001, r=0.61, CI (0.47 to 0.72)) was observed in group 2.

**Conclusion:** During phacoemulsification, surge events are more likely to occur when operating at high IOP settings.

**Abbreviations:** LIPMICS = low infusion pressure microincision cataract surgery, IOP = intraocular pressure, CDE = cumulative dissipated energy, UJS = mean ultrasound time, AS = Active sentry, LOCS = Lens Opacities Classification System, NO = nuclear opalescence, AFR = aspiration flow rate

## Introduction

Cataract is one of the leading causes of blindness worldwide in adults aged over 50 years and older, causing approximately 45% of 33.6 million cases of global blindness in 2020 [**1**]. 

Since the revolutionary development of phacoemulsification by Charles Kelman in 1967, many enhancements were made to improve surgical outcome, efficacy, and safety profile. In recent years, phaco fluidics have been the subject of numerous studies to improve the performance of the device and raise the predictability of phacoemulsification [**2**,**3**].

All phacoemulsification machines essentially have the same components and basic concepts. Phacoemulsification makes use of ultrasound technology, as well as vacuum, while continuously balancing fluidics within the eye.

During phacoemulsification, if the phaco tip becomes fully occluded by lens material, there is a consequent vacuum buildup until the preset vacuum limit is reached. When the occlusion is broken (lens fragment is emulsified/ dislodged) and fluid rushes from the anterior chamber (positive pressure, smaller volume) towards the pump/ cassette (negative pressure, higher volume) causing a subsequent drop in the IOP. This secondary effect is called postocclusion break or surge. IOP fluctuation during surge can lead to a shallow anterior chamber or iris/ posterior capsule trauma [**4**].

In traditional phacoemulsification systems, the infusion bottle height or pressurized infusion level is preset to balance irrigation and aspiration and to reduce the effect of surge. Continuous reduction in main incision size over the last two decades required an increased infusion pressure causing non-physiologically high IOP and substantial intraoperative IOP fluctuations [**5**].

Recently, a new fluidics management system (Centurion Vision System, Alcon Laboratories, USA), that actively manages irrigation, has been introduced. This system was later updated with an IOP pressure sensor in the phaco handpiece (Active Sentry Handpiece, Alcon Laboratories, USA) to improve performance and hasten response to surge. Each time the active sentry is activated, it prevents or mitigates a surge event [**6**]. For the first time, this allows surgeons to perform phacoemulsification at physiological IOP levels.

The aim of our study was to compare the number of surge events and efficacy of phacoemulsification using a near physiological IOP setting and a standard IOP setting.

## Materials and methods

The surgical data of 181 patients who underwent phacoemulsification with IOL implantation using the CENTURION Vision System’s (Alcon) Active Fluidics setting and Active Sentry Handpiece (Alcon) were retrospectively analyzed. 

**Fig. 1:**
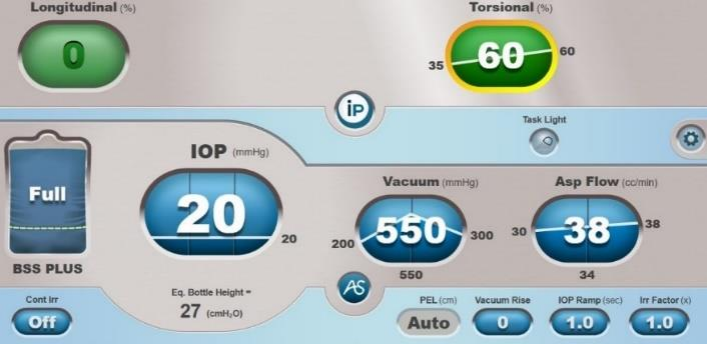
Phacoemulsification settings

All surgeries were carried out by the same surgeon (G.S.) between March 2020 and February 2021. Using the Active Fluidics, a target IOP was set at near physiological or standard pressure (20 mmHg and 60 mmHg, respectively). The patients’ age, sex, perioperative slit lamp cataract grading using the Lens Opacities Classification System (LOCS) III, vacuum and aspiration rate setting, as well as the CENTURION Vision System’s (Alcon) overall case report were recorded. Phacoemulsification fluidics settings and consumables are summarized in **Table 1** (**Fig. 1** showing phaco fluidics settings).

Statistical analysis was conducted using the SPSS program (IBM SPSS Statistics 26). Differences were tested by the two-tailed t-test. Pearson’s correlation was used to analyze the association between all the studied parameters. Data were expressed as mean ± standard deviation and results were considered as statistically significant when the p-value was smaller or equal to α (α=0.05).

**Table 1:** Phaco fluidics and consumables

Cataract surgery settings	Group I	Group II
Phacoemulsification tip	Intrepid balanced tip (Alcon Laboratories, USA)	Intrepid balanced tip (Alcon Laboratories, USA)
Incision size (mm)	2.2	2.2
Phacoemulsification settings for aspiration of the quadrants (conquer)		
Power (torsional, max)	60%	60%
Aspiration flow rate	30-38 ml/ min	30-38 ml/ min
Vacuum (peak setting)	200-550-300 mmHg	200-550-300 mmHg
IOP setting	20 mmHg	60 mmHg

## Results

In total, 204 eyes of 181 patients were considered for the study. Patients were categorized into two groups: in the first group the target IOP was set at 20 mmHg (n=102), whereas in the second group a 60-mmHg setting was used (n=102). Patient demographics and cataract characteristics were similar in both sample groups. Cataracts were subdivided into nuclear opalescence (NO) grades 2-4 according to the LOCS III. In the first group, 48% (n=49) were LOCS 2, 42.2% (n= 43) were LOCS 3 and 9.8% (n=10) were LOCS 4 cataracts, whereas in the second group, 47.1% (n=48) were LOCS 2, 43.1% (n=43) were LOCS 3 and 9.8% (n=10) were LOCS 4 cataracts. The mean age in the first group was 71.1 ± 10.1 years and 71.6 ± 10.5 years, in the second group. In the first group, 58,8% of the patients were female (n=60) and 41,2% were male (n=42), whereas in the second group, 63,7% of the patients were female (n=65) and 36,3% were male (n=37).

The total case time, meaning from the beginning of the infusion until the end of the Irrigation/ Aspiration, was significantly lower (p=.036) when the IOP was set at 20 mmHg, with an average duration of 0:03:17.20 ± 0:00:34.55, compared to the 60-mmHg setting, in which the average duration was 0:03:28.71 ± 0:00:43.03.

The mean cumulative dissipated energy (CDE) during phacoemulsification was 7.06 ± 3.20 in the first group, and 7.59 ± 3.26 in the second group, showing no statistically significant difference between the two settings (p=0.24). The total ultrasound time (UJS time) was on average 0:00:36 ± 0:00:12 in the first group and 0:00:38 ± 0:00:13 in the second group, showing no statistically significant difference between the two groups (p=0.31). 

During nucleus removal, active sentry (AS) engaged less often (p<0.001) when the target IOP was set at 20 mmHg, compared to the 60-mmHg setting. Furthermore, in the cases in which AS was activated, the total number of AS actuations was significantly lower in the first group (**Table 2**). No statistically significant correlation between the CDE and AS activation was observed in the first group (p=0.96). In the second group, a statistically significant correlation was observed between the CDE and AS actuations (p<0.0001). Pearson’s correlation showed a strong positive relationship between the two variables, r=0.61, CI (0.47-0.72).

**Table 2:** AS actuation on 20 mmHg and 60 mmHg setting

AS Actuations		0	1	2	3	4	5	7	9	11	Total
Target IOP (mmHg)	20	78	9	5	4	3	3	0	0	0	102
	60	45	19	15	11	1	5	1	3	2	102
Total		123	28	20	15	4	8	1	3	2	204

The average aspiration time was 0:02:00.74 ± 0:00:25.54 in the first group, and 0:02:11.25 ± 0:00:25.82 in the second group, whereas the total fluid consumption during aspiration was estimated to be 32.90 ± 7.67 vs. 37.16 ± 8.50, respectively. The aspiration time and the estimated fluid consumption were both significantly lower (p = .004 and p < 0.001, respectively) when the target IOP was set at 20 mmHg.

## Discussion

Two randomized controlled trials previously demonstrated that the active-fluidics Centurion® phacoemulsification system is more efficient than the gravity-fluidics Infiniti® IP system for cataracts with similar morphological characteristics. The studies showed that the CDE count, aspiration time and estimated fluid consumption were significantly lower using the active-fluidics configuration compared to the gravity-fluidics configuration [**7**,**8**]. In our case, the CDE showed no statistically significant difference between the two IOP settings (20 vs. 60 mmHg). As the active fluidics technology ensures a constant IOP, the CDE is mostly influenced by the aspiration flow rate (AFR) and the vacuum at a given lens hardness. As the vacuum and AFR settings were relatively constant in both groups, the IOP setting alone did not affect the CDE significantly. In contrast, we noticed a statistically significant reduction in the aspiration time and the estimated fluid consumption with the 20-mmHg setting. The aspiration time took an average of 11 seconds less and approximately 11.5% less fluid was used for the surgery. 

It is already known that surge events are more likely to occur on a higher vacuum and aspiration rate setting, but there is little information on how the preset intraocular pressure affects surge. A laboratory study on a 3-spring eye model compared volumetric occlusion break surge responses during phacoemulsification on different vacuum and IOP settings on 3 generations of Alcon phacoemulsification units. The study concluded that occlusion break surge volumes decreased with decreasing vacuum limit and increasing target IOP [**9**]. 

**Fig. 2:**
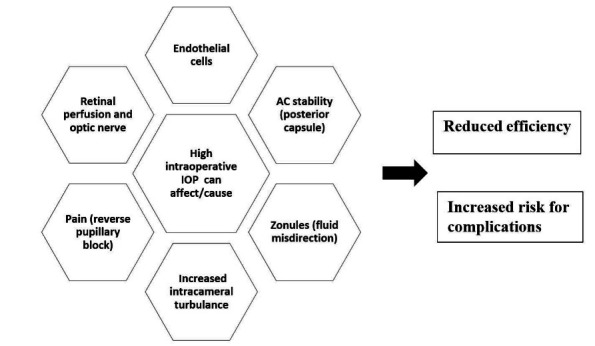
Consequences of high intraoperative IOP

Vasavada et al. compared the impact of varying fluidic parameters on intraoperative IOP fluctuations and postoperative outcomes. They concluded that higher aspiration flow rate and bottle heights are associated with high intraoperative IOPs and prolonged elevated IOPs during cataract surgery, leading to greater anterior segment inflammation [**5**]. Vasavada et al. also examined the anterior vitreous face during phacoemulsification using different AFR and target IOP settings on porcine eyes. They reported 2 cases of ruptured anterior vitreous face in the higher fluidic settings (AFR = 40 cc/ min, bottle height = 110 cm, vacuum = 650 mm Hg) [**10**]. Hejsek et al. evaluated IOP fluctuations and calculated the mean ocular perfusion pressure during phacoemulsification on patients. On a 100 cm bottle height (72 mmHg), they found that the maximum ocular perfusion pressure was higher than 50 mmHg and 60 mmHg in 89% and 30% of the cases, respectively. Such high pressures may induce impairment of perfusion of the optic nerve, retina, and choroid [**11**]. A recent study of acute ocular hypertension on a mouse model, that mimics a transient IOP elevation during phacoemulsification, concluded that operating at a higher intraocular pressure (90 mmHg setting vs. 45 mmHg setting) causes cellular and molecular retinal injuries [**12**]. All these previous studies suggest that operating on high bottle height/ target IOP settings can negatively affect the anatomy and physiology of the eye (**Fig. 2**). This risk might be even higher in pre-damaged eyes.

**Fig. 3:**
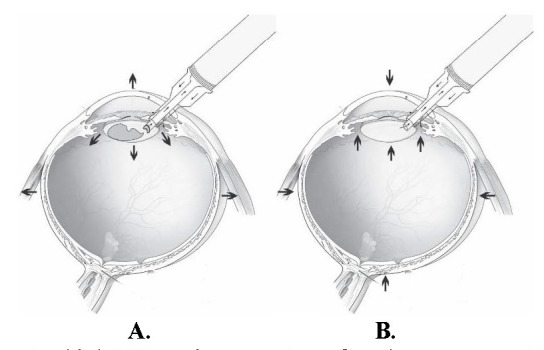
**A.** Operating under high IOP settings, tension of ocular structures. **B.** Surge, shallow AC, possible iris/ corneal trauma

Our results showed that surge events occur significantly less often, when operating at a physiological IOP setting (20 mmHg) and when it occurs, the number of surge events are also lower compared to the standard IOP setting (60 mmHg). We assumed, that when operating at physiological IOP settings, the anterior chamber is more stable during the surgery, which makes the operation safer and (potentially) easier. We theorized that surge events are less likely to occur when operating at low IOP settings, because the pressure gradient, which the irrigation should compensate during an occlusion break, is lower. Furthermore, we hypothesized, that operating at high IOP settings leads to more frequent surge events due to the additional tension on ocular structures like the cornea, sclera, vitreous and capsule (**Fig. 3**). In addition, according to our findings, the number of surge events also rises at a high target IOP with the rising energy consumption. One could argue that the pressure sensor in the active sentry handpiece is not sensitive enough at such low pressures to register sudden changes in the IOP. However, had this been the case, we would have constantly been tackling anterior chamber instability, which did not occur.

## Conclusion

In this study, we compared the influence of the target IOP settings during phacoemulsification using the same phaco unit with the same vacuum and AFR settings. Our results showed that operating at physiological IOP setting is faster and possibly safer than operating at an elevated IOP setting.

To our knowledge, this is the only phaco machine that allows surgeons to operate at such low target IOP. In our daily clinical practice, we prefer to operate at near physiological IOP settings independent of cataract stage and ocular comorbidities. We believe that the results of our study allow surgeons to reconsider their intraoperative target IOP to reduce the number of surge events and therefore increase the safety of cataract surgery.

**Conflict of Interest Statement**

Prof. Dr. Med. Gabor B. Scharioth is a consultant for Alcon Laboratories, Inc. and Medicontur Medical Engineering Ltd., Inc.

**Informed Consent and Human and Animal Rights statement**

Informed consent has been obtained from all the patients included in the study.

**Authorization for the use of human subjects**

The study was conducted according to the guidelines of the Declaration of Helsinki. As the study was retrospective, the Institutional Board decided that we did not need an ethical approval number.

**Acknowledgements**

None.

**Sources of Funding**

This research received no external funding.

**Disclosures**

None. 

**Data availability**

All data were fully anonymized and are available upon request.
